# Sulbactam-durlobactam combination therapy for carbapenem-resistant *Acinetobacter baumannii* sepsis with cutaneous involvement in an infant with acute monocytic leukemia: A case report

**DOI:** 10.3389/fphar.2025.1633982

**Published:** 2025-09-04

**Authors:** Chunxi Fu, Xue Tang, Xia Guo, Liang Huang, Ju Gao, Rong Yang

**Affiliations:** ^1^ Department of Pediatrics, West China Second University Hospital, Sichuan University, Chengdu, China; ^2^ Key Laboratory of Birth Defects and Related Diseases of Women and Children, Ministry of Education, Sichuan University, Chengdu, China; ^3^ Department of Pharmacy, West China Second University Hospital, Sichuan University, Chengdu, China; ^4^ Evidence-Based Pharmacy Center, West China Second University Hospital, Sichuan University, Chengdu, China

**Keywords:** carbapenem-resistant *Acinetobacter* baumannii, sulbactam-durlobactam, infant, sepsis, case report

## Abstract

Carbapenem-resistant *Acinetobacter baumannii* (CR*Ab*) causes severe infections and poses significant treatment challenges. Sulbactam-durlobactam (SUL-DUR) is a novel antibiotic, but its use in children and infants has not been reported. We reported the first case of an infant with acute leukemia successfully treated with SUL-DUR for CR*Ab* sepsis with cutaneous involvement.

## Introduction

*Acinetobacter baumannii* infection is regarded as one of the most difficult bacterial infections to control, especially with the global spread of carbapenem-resistant *Acinetobacter baumannii* (CR*Ab*) in the past two decades (Vrancianu et al., 2020). Studies have demonstrated that, compared with carbapenem-sensitive *A. baumannii* infections, patients infected with drug-resistant strains face a more than twofold increase in mortality risk (Falcone et al., 2023).

Durlobactam (formerly known as EXT2514) is a β-lactamase inhibitor that can effectively inhibit the hydrolysis of sulbactam by class A, C and D enzymes produced by CRAb (Findlay et al., 2022). Under the synergistic protection of Durlobactam, a low dose of sulbactam can still effectively act on the penicillin-binding protein (PBP) target (Findlay et al., 2022). In a study encompassing 246 CR*Ab* strains, the combined use of Durlobactam and sulbactam significantly enhanced its antibacterial efficacy, reducing the minimum inhibitory concentration (MIC) of SUL by 16 to 64 folds (Karlowsky et al., 2022). In 2023, the US FDA approved sulbactam-durlobactam (SUL-DUR) for treating pneumonia caused by CR*Ab* in patients aged 18 years or older (Food and Drug, 2023). Studies indicate that SUL-DUR is non-inferior to colistin in efficacy for CR*Ab* infections and exhibits a significantly lower nephrotoxicity rate compared to colistin (Kaye et al., 2023).

To our knowledge, there are currently no published reports on the use of SUL-DUR for treating CR*Ab* infections in paediatric. Moreover, the vast majority of current studies mainly concentrate on cases of pneumonia and sepsis, while there is a dearth of explicit research data regarding the therapeutic effect of SUL-DUR in treating skin and soft tissue CR*Ab* infections. Herein, we present the first reported case of successful SUL-DUR-based combination therapy for CR*Ab* sepsis with cutaneous involvement in an infant with leukemia.

## Case report

**FIGURE 1:**
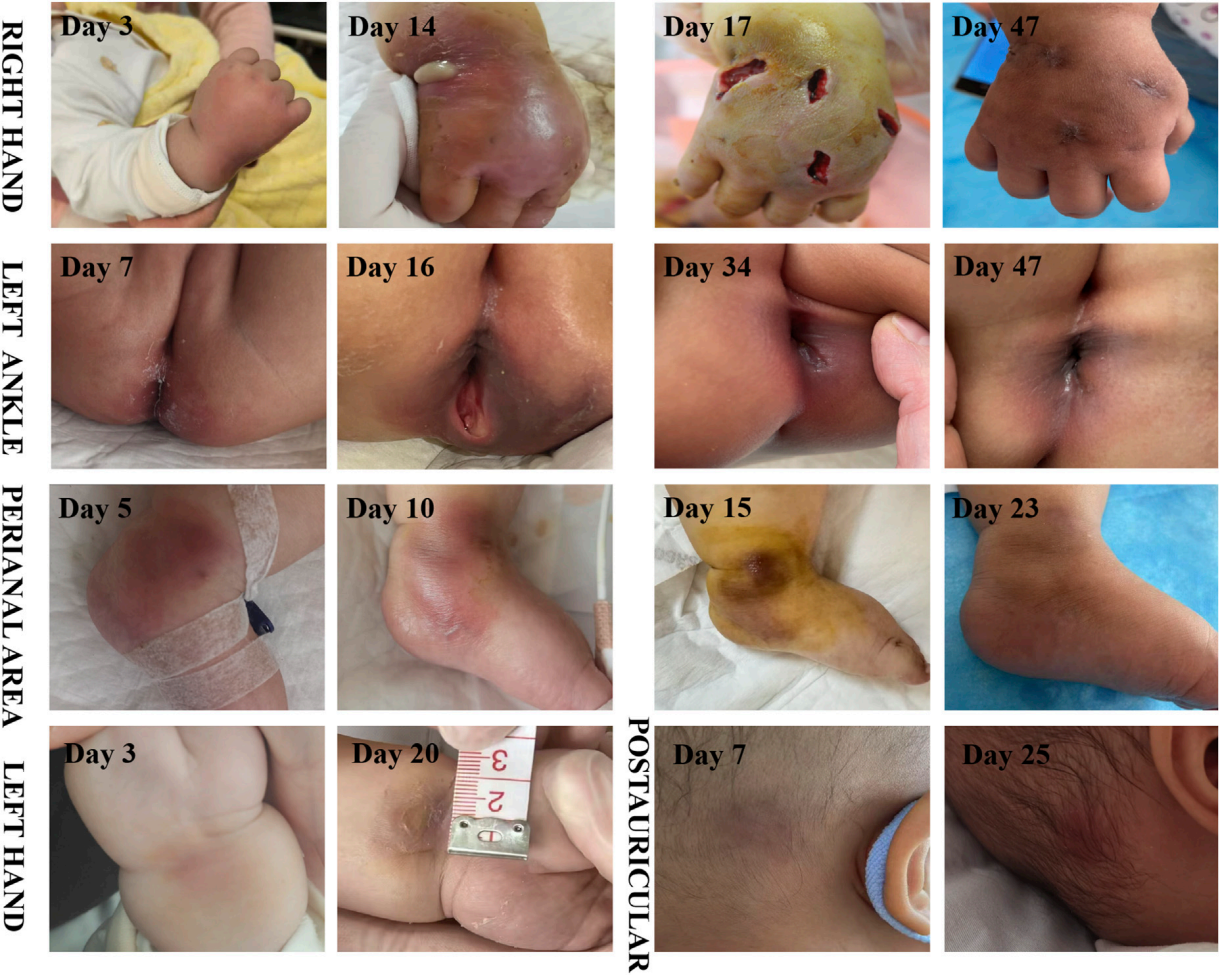
Time course of skin abscess (The day of the first occurrence of fever is regarded as the first day).

**FIGURE 2:**
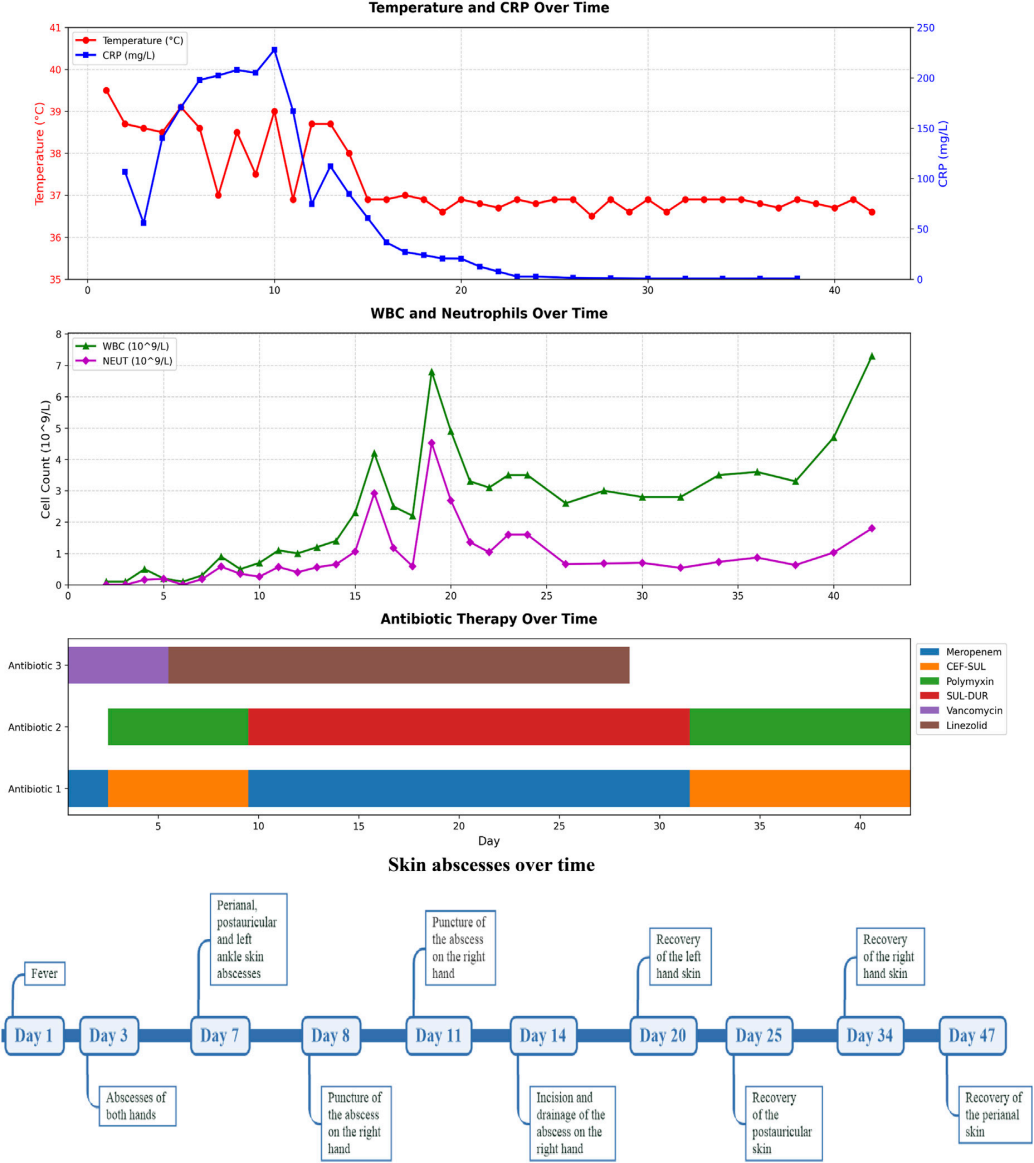
Timeline diagram of disease and treatment course. CRP, C-reactive protein; WBC, White Blood Cell; NEUT, Neutrophil; SUL-DUR, sulbactam-durlobactam; CEF-SUL, Cefoperazone-Sulbactam.

A 10-month-old female infant was admitted to the hospital 3 months ago due to skin ecchymosis and subcutaneous nodules, and was diagnosed with acute monocytic leukemia. After the first course of chemotherapy, the bone marrow achieved a complete remission state. There has been a history of two previous episodes of invasive mechanical ventilation. This time, during the bone marrow suppression period after the third course of chemotherapy, the patient manifested symptoms of high fever, chills, and cold extremities. Empirical antibacterial therapy was immediately initiated with meropenem, vancomycin, and voriconazole. Peripheral blood and blood samples from the peripherally inserted central catheter were collected for culture. About 10 h after the blood sample was collected, the blood culture results from both sides indicated *A. baumannii*. Meanwhile, a systematic screening for systemic infection foci was carried out on the patient to eliminate potential infection sources. The results indicated that the patient presented no symptoms such as diarrhea, abdominal pain, dysuria, or cough. No obvious infection foci were detected in the lung imaging examination. The echocardiography suggested no significant vegetations on the heart valves. The metagenomic pathogen detection of the cerebrospinal fluid yielded a negative result. About 48 h culture results indicated CR*Ab*, and gene sequencing further verified that the strain carried the OXA-23 resistance gene. The specific drug susceptibility results are presented in Table 1. Metagenomic sequencing revealed 273,311 specific sequences of *A. baumannii*. Based on the above findings, the antibiotic treatment was adjusted to polymyxin B (20,000 units/kg per dose, twice daily) in combination with a high-dose of cefoperazone sulbactam (dose of sulbactam was 80 mg/kg per dose, every 8 h), and the PICC catheter was removed. Despite the administration of polymyxin B plus high-dose cefoperazone-sulbactam for 1 week, the infant persisted in having a fever. Worse still, multiple skin infections occurred and progressively aggravated (Figure 1). CR*Ab* with the same resistance profile as that in the blood culture was also isolated from the culture of the puncture fluid of the right hand abscess. Following comprehensive discussions with the patient’s family, the antibiotic regimen was modified to incorporate the off-label, compassionate use of SUL-DUR (200 mg/kg/day of the sulbactam component, every 6 h, with an infusion duration of more than 3 h) in combination with meropenem (40 mg/kg per dose, every 8 h). After the modification of the treatment, the infant’s body temperature gradually returned to normal and the CRP level decreased significantly (Figure 2). Subsequently, we conducted additional SUL-DUR-related drug susceptibility assays. As depicted in Supplementary Figure S1, determined via the KB method, SUL-DUR exhibited sensitivity towards CR*Ab* in blood culture specimens and pus culture specimens (with an inhibition zone diameter of 25 mm).

**TABLE 1:** Antimicrobial susceptibility of the OXA-23-producing *Acinetobacter baumannii* isolate obtained from blood and pus.^a^

Antimicrobial	Method	Result	Unit	Susceptibility	Interpretive ranges
Piperacillin-tazobactam	MIC	≥128.0	μg/mL	R	16–128
Cefoperazone-sulbactam	MIC	32.0	μg/mL	I	
Ticarcillin-Clavulanic	MIC	≥128.0	μg/mL	R	16–128
Cefepime	MIC	≥32.0	μg/mL	R	8–32
Ceftazidime	MIC	≥64.0	μg/mL	R	8–32
Aztreonam	MIC	≥64.0	μg/mL	R	
Amikacin	MIC	≥64.0	μg/mL	R	16–64
Imipenem	MIC	≥16.0	μg/mL	R	4–16
Meropenem	MIC	≥16.0	μg/mL	R	4–16
Tobramycin	MIC	≥16.0	μg/mL	R	4–16
Ciprofloxacin	MIC	≥4.0	μg/mL	R	1–4
Levofloxacin	MIC	≥8.0	μg/mL	R	2–8
Sulfamethoxazole-trimethoprim	MIC	≥16.0/304.0	μg/mL	R	2–4
Colistin	MIC	≤0.5	μg/mL	S	2–4
Minocycline	MIC	≥16.0	μg/mL	R	4–16
Doxycycline	MIC	≥16.0	μg/mL	R	4–16
Tigecycline	MIC	≥8.0	μg/mL	R	1–4
Piperacillin	KB	6	mm	R	
Gentamicin	KB	6	mm	R	12–15
Fosfomycin	Etest	≥512.0	μg/mL	R	
Ceftazidime-avibactam	Etest	256	μg/mL	R	

Abbreviations:R, resistant; I, intermediate; S, sensitive; MIC, minimum inhibitory concentration; KB, Kirby-Bauer.

Note: Antimicrobial susceptibility testing was performed using a modified Kirby-Bauer disc diffusion method. After cultivation, the bacterial suspension was inoculated onto Mueller-Hinton agar plates, and standard antibiotic discs were used for testing. The interpretive breakpoints for antimicrobial susceptibility were based on CLSI, and EUCAST, standards, which are regularly updated.

^a^ Two blood cultures and one pus culture each yielded the same OXA-23-producing *Acinetobacter baumannii* isolate with identical antimicrobial susceptibility patterns.

Systemic infection of the skin were getting better, except for the right hand. On the 14th day of the infection, an incision and drainage procedure was performed on the right-hand abscess, due to obvious swelling and a fluctuating sensation in the local skin area. The culture of the pus from the right hand abscess also confirmed *A. baumannii* carrying the OXA-23 gene.

Throughout the SUL-DUR treatment period, no antibiotic-related adverse events or infection recurrences occurred. After 21 days of continuous treatment with SUL-DUR in combination with meropenem, metagenomic sequencing revealed 3 specific sequences of *A. baumannii*. The antibiotic regimen was adjusted to polymyxin B combined with cefoperazone sulbactam for an additional 13 days of treatment. Re-examination of pathogen metagenomic sequencing failed to detect *A. baumannii*, and all antibiotics were discontinued. After 1 month of anti-infective treatment, the infant underwent allogeneic hematopoietic stem cell transplantation successfully. Throughout the subsequent follow-up period, no adverse reactions or recurrence of infections were observed.

Note: The drug dosages for treating *Acinetobacter baumannii* infection are as follows: Polymyxin B (20,000 units/kg per dose, twice daily); SUL-DUR (200 mg/kg/day of the sulbactam component, every 6 h, with an infusion duration of more than 3 h); CEF-SUL (dose of sulbactam was 80 mg/kg per dose, every 8 h); Meropenem (40 mg/kg per dose, every 8 h).

## Discussion

To our knowledge, this is the first reported case of successful SUL-DUR treatment for CR*Ab* infection in an infant and the first use of SUL-DUR as salvage therapy for agranulocytosis-associated sepsis with cutaneous involvement caused by CR*Ab*. This success highlights the importance of SUL-DUR in severe CR*Ab* infections. Administered at guideline-recommended doses, SUL-DUR showed no adverse effects, confirming its safety and efficacy in infants. This is particularly valuable when other antibiotics like polymyxin, cefoperazone-sulbactam, or tigecycline are less effective.

Bloodstream infections caused by CR*Ab* exhibit a 30-day mortality rate of up to 42% (Wang et al., 2024), with infant mortality from Acinetobacter-positive sepsis reaching 59% (Collaboration, 2016). Currently, the therapeutic options for CR*Ab* infections are limited, particularly in pediatric and infant populations, polymyxin B or tigecycline remain the most frequently used drugs in clinical practice (Shields et al., 2023; Lockowitz et al., 2025). In our case, the MIC of cefoperazone-sulbactam against CR*Ab* was 32, which was classified as intermediate. Initially, we used a high dose of cefoperazone sulbactam based on the drug sensitivity test results, attempting to treat the patient’s CR*Ab* sepsis by enhancing β-lactamase inhibition and additional binding to PBP3 at high concentrations. However, after 1 week of treatment, there was no improvement in body temperature and inflammatory indicators, so we switched to SUL-DUR as a salvage treatment. We speculate that the poor efficacy of high-dose cefoperazone sulbactam may be due to the following reasons: CR*Ab* itself has strong resistance to this drug. Studies have shown that when the MIC of cefoperazone is ≥ 16 μg/mL, CR*Ab* patients can be divided into two groups: in the cefoperazone-sulbactam sensitive group, the in-hospital mortality rate of patients treated with high-dose cefoperazone sulbactam combined with colistin or other antibiotics is still as high as 39%; while in the insensitive group, the mortality rate of patients treated with colistin combined with other antibiotics is even higher, reaching 68% (Kanchanasuwan et al., 2021). Another study on extensively drug-resistant *A. baumannii* also showed that only 62.5% of patients responded effectively to cefoperazone-sulbactam treatment (Li et al., 2015). Although the combination of this drug and colistin has certain antibacterial activity against CR*Ab in vitro*, it still faces many challenges in clinical practice. Secondly, this infant patient was in the bone marrow suppression period, with extremely low immune function and a neutrophil count of 0, which may further weaken the treatment response to cefoperazone sulbactam and colistin. Durlobactam can inhibit the degradation of sulbactam by class A, B and D enzymes produced by CR*Ab*, thereby providing additional protection for beta-lactamase inhibitors that ampicillin or cefoperazone cannot achieve (Findlay et al., 2022; Tamma et al., 2024), which may be the reason why SUL-DUR shows better efficacy in clinical practice. In addition, *in vitro* studies have shown that combining carbapenems with SUL-DUR can protect carbapenems from degradation by OXA-carbapenemases, thereby enabling more effective and complementary inactivation of multiple PBPs(Veeraraghavan et al., 2025).

The Infectious Diseases Society of America document, “Recommended Doses of Selective β-Lactam Antibiotics for the Treatment of Antimicrobial-Resistant Gram-Negative Bacterial Infections in Children,” released in 2025, further indicated that SUL-DUR in combination with meropenem or imipenem may be effective for CR*Ab* infections (Lockowitz et al., 2025). Nevertheless, the guideline highlighted the current lack of research data regarding the use of SUL-DUR in infants and children (Lockowitz et al., 2025). This case describes an infant with severe post-chemotherapy bone marrow suppression, multiple skin infections, and abscesses. Despite early treatment with meropenem, polymyxin, high-dose cefoperazone-sulbactam, and surgical drainage, fever and elevated CRP persisted. Clinical cure and microbiological clearance were achieved only after introducing SUL-DUR combined with meropenem. During the treatment process, we monitored the clinical symptoms, signs and blood routine indicators of the patients daily, and tested liver and kidney functions and electrolyte levels every other day. No adverse reactions related to SUL-DUR were observed, suggesting that this drug may also have good safety in the infant and toddler population.

*A*. *baumannii* is not a common pathogen for skin and soft tissue infections (Ray et al., 2013). However, due to its frequent multi-drug resistance and the propensity to be complicated with bacteremia, it leads to a higher mortality rate (Guerrero et al., 2010). Excluding cases related to gunshot wounds or natural disasters, *A. baumannii* skin and soft tissue infections predominantly occur in specific populations, such as HIV-infected individuals, immunosuppressed patients, and those with liver cirrhosis (Charnot-Katsikas et al., 2009; Guerrero et al., 2010). The summary of related cases is presented in Table 2. In the 18 cases of *A. baumannii* skin and soft tissue infections, 61.1% of the patients died. Among the 13 patients with *A. baumannii* bacteremia, the mortality rate was as high as 84.6%. Similar to this case, Pereira *et al.* (Pereira et al., 2019) documented two patients, namely, one with acute promyelocytic leukemia and the other with Burkitt’s lymphoma. Both of them, during the myelosuppression period following chemotherapy, manifested facial cellulitis. Despite undergoing aggressive anti-infective therapy encompassing vancomycin, meropenem, tigecycline, and polymyxin, both patients ultimately succumbed to septic shock induced by *A. baumannii*. In contrastat the early stage, the patient presented solely with sepsis, without any signs of skin and soft tissue involvement. As the disease progressed, multiple cutaneous infection foci developed. After the antibiotic regimen was adjusted to SUL-DUR, the cutaneous infections in other areas showed some remission. However, the abscess on the right hand continued to increase in size. Both the pus culture and the metagenomic sequencing results confirmed that the skin infection of the infant was caused by CR*Ab* with the same resistance profile as that in the blood culture. After treatment with SUL-DUR combined with meropenem and incision and drainage of the most severely infected right hand, the infant’s multiple skin and soft tissue infections were effectively controlled. This positive outcome can be attributed not only to the administration of effective antibiotics but also to the timely intervention of surgical drainage.

**TABLE 2:** Characteristics of *Acinetobacter* baumannii skin and soft tissue infections associated with non-gunshot wounds and non-natural disaster scenarios.

Case	Age	Sex	Underlying conditions	Carbapenem-resistant	*Acinetobacter* bacteremia	Surgical intervention	Treatment	Outcome	Ref
1	12	M	Acute promyelocytic leukemia, myelosuppression period	Y	Y	N	Tigecycline and polymyxin	Died	Pereira et al. (2019)
2	10	M	Burkitt lymphoma, myelosuppression period	Y	Y	N	Meropenem and polymyxin	Died	Pereira et al. (2019)
3	75	M	Percutaneous endoscopic gastrostomy, chronic kidney disease	N	Y	Y	Colistin and linezolid	Survival	Guerrero et al. (2010)
4	50	M	Morbidly obese, Chronic leg ulcer	N	Y	N	Ampicillin-sulbactam	Survival	Guerrero et al. (2010)
5	50	M	Cirrhosis	N	Y	Y	Ertapenem	Died	Guerrero et al. (2010)
6	16	F	Traumatic transfemoral amputation of both legs together	Y	N	N	Colistin, meropenem and rifampin	Survival	Biancofiore et al. (2007)
7	35	F	Paranoid schizophrenia	Y	N	N	Ampicillin-sulbactam and meropenem	Survival	Hiraki et al. (2013)
8	83	M	Cirrhosis	Unknown	Y	Unknown	Unknown	Died	Sullivan et al. (2010)
9	41	M	Morbidly obese, cirrhosis	N	Y	Y	Ciprofloxacin, meropenem and lincomycin	Died	Ali et al. (2014)
10	61	M	Cirrhosis	N	Y	Y	Meropenem	Died	Nonaka et al. (2014)
11	48	F	HIV	Y	Y	Y	Vancomycin and aztreonam	Died	Sinha et al. (2014)
12	54	M	N	Y	N	Y	No (Resistant to all antibiotics)	Survival	Ramampisendrahova et al. (2020)
13	50	F	N	Y	N	N	Tigecycline	Survival	Adler et al. (2014)
14	49	M	Diabetes, pancreas-kidney transplant recipient	Y	Y	Y	Imipenem, vancomycin, and polymyxin	Died	Clemente et al. (2012)
15	21	M	SLE, using immunosuppressants, exploratory laparotomy	Y	Y	Y	Imipenem, amikacin, vancomycin, and clindamycin	Died	Charnot-Katsikas et al. (2009)
16	47	F	HIV, exploratory laparotomy	Y	Y	Y	Piperacillin-tazobactam, vancomycin, and colistin	Died	Charnot-Katsikas et al. (2009)
17	37	F	SLE, using immunosuppressants	Y	Y	Y	Levofloxacin	Died	Matthews et al. (2019)
18	73	F	rheumatoid arthritis, using immunosuppressants	N	N	Y	Ciprofloxacin	Survived	Lai et al. (2023)

SLE, systemic lupus erythematous; HIV, human immunodeficiency virus; N, no; Y, yes; F, female; M, male.

Nevertheless, regrettably, due to the relatively scarce clinical application of SUL-DUR in China, the precise determination of its distribution concentration in pus remains currently unfeasible.

Although this case report provides the first evidence of the potential efficacy of SUL-DUR in the treatment of sepsis and skin involvement caused by CR*Ab* in infants, there are still certain limitations. Firstly, as this study is a case report, the generalizability of its results is somewhat limited. Secondly, although the patient was switched to a SUL-DUR combined with meropenem regimen after the failure of high-dose cefoperazone-sulbactam combined with polymyxin, due to the lack of a concurrent control group, it is still difficult to directly compare the efficacy and safety of the two treatment regimens in CR*Ab* infections. In addition, this study has not provided a systematic safety assessment or long-term follow-up data. Therefore, it is necessary to conduct larger sample size, well-designed randomized controlled clinical trials to further evaluate the efficacy and safety of SUL-DUR-based antibacterial treatment regimens in infant CR*Ab* infections, especially in vulnerable populations with a higher demand for antibiotics that exhibit lower nephrotoxicity and enhanced safety.

### The patient’s perspective

We hereby report the following information regarding the patient’s experience: “Following chemotherapy, my daughter developed a severe infection, which could not be effectively controlled by conventional antibiotic therapies. After comprehensive consultations with physicians and pharmacists, it was determined to initiate treatment with the novel antibacterial agent, SUL-DUR. Post - administration of SUL-DUR, a notable improvement was observed in both my daughter’s febrile symptoms and multiple abscesses across the body. Throughout the course of treatment, no significant discomfort or adverse reactions were detected, and I am satisfied with the therapeutic efficacy. We are very thankful for the medical team’s knowledge and continue to hope that this therapy might assist other families in similar situations.

## Conclusion

This case report presents an infant diagnosed with leukemia who underwent chemotherapy. During the bone marrow suppression phase, the infant developed sepsis with cutaneous involvement caused by CR*Ab*. Despite treatment with meropenem, polymyxin, and cefoperazone-sulbactam, the infections remained uncontrolled. Subsequently, treatment with SUL- DUR was initiated, which successfully managed the infections. It is important to note that there are currently no published reports on the use of SUL-DUR in patients younger than 18 years of age. This single case suggests that SUL-DUR may be an effective treatment option for CR*Ab* infections in infants, with no apparent adverse reactions noted. While our findings provide preliminary evidence supporting the potential efficacy of SUL-DUR in paediatric populations, further research is necessary to establish its clinical utility. Larger-scale efficacy trials with extended follow-up periods are required to confirm these preliminary results. Importantly, paediatric-specific pharmacokinetic studies and comprehensive safety evaluations in younger age groups should be prioritized, as current data remain limited for this vulnerable patient population.

## Data Availability

The original contributions presented in the study are included in the article/Supplementary Material, further inquiries can be directed to the corresponding author.

## Abbreviations

CRAb, carbapenem-resistant *Acinetobacter* baumannii; SUL-DUR, sulbactam-durlobactam; CRP, C-reactive protein; WBC, White Blood Cell; NEUT, Neutrophil; SLE, Systemic lupus erythematous; HIV, human immunodeficiency virus.
