# Supplementary figures and images for: Sulbactam-durlobactam combination therapy for carbapenem-resistant Acinetobacter baumannii sepsis with cutaneous involvement in an infant with acute monocytic leukemia: A case report
Source: Front Pharmacol. 2025 Sep 4;16:1633982. doi: 10.3389/fphar.2025.1633982 (PMC12443841; doi:10.3389/fphar.2025.1633982)

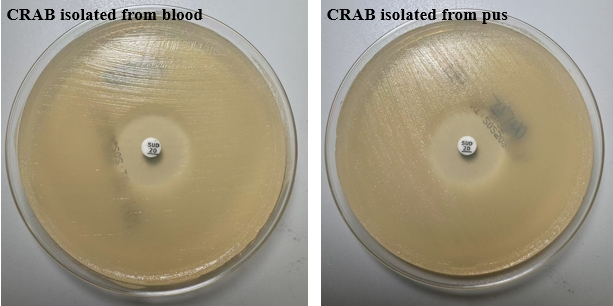

Supplement: Supplementary file 1 [file Image1.tif]
